# Elementary immunology: Na^+^ as a regulator of immunity

**DOI:** 10.1007/s00467-016-3349-x

**Published:** 2016-02-26

**Authors:** Valentin Schatz, Patrick Neubert, Agnes Schröder, Katrina Binger, Matthias Gebhard, Dominik N. Müller, Friedrich C. Luft, Jens Titze, Jonathan Jantsch

**Affiliations:** 1 Institute of Clinical Microbiology and Hygiene, Universitätsklinikum Regensburg–Universität Regensburg, Franz-Josef-Strauß-Allee 11, 93053 Regensburg, Germany; 2 Department of Nephrology and Hypertension, Universitätsklinikum Erlangen–Friedrich-Alexander Universität (FAU) Erlangen-Nürnberg, Erlangen, Germany; 3 Baker IDI Heart and Diabetes Institute, Melbourne, Australia; 4 Experimental and Clinical Research Center (ECRC), Research Building, Charité Lindenberger Weg 80, Berlin, Germany; 5 Max-Delbrück Center for Molecular Medicine, Berlin, Germany; 6 Division of Clinical Pharmacology, Department of Medicine, Vanderbilt University School of Medicine, Nashville, TN USA

**Keywords:** Local Na^+^ availability, Skin salt storage, Macrophages, T cells, Immune cell function and activation

## Abstract

The skin can serve as an interstitial Na^+^ reservoir. Local tissue Na^+^ accumulation increases with age, inflammation and infection. This increased local Na^+^ availability favors pro-inflammatory immune cell function and dampens their anti-inflammatory capacity. In this review, we summarize available data on how NaCl affects various immune cells. We particularly focus on how salt promotes pro-inflammatory macrophage and T cell function and simultaneously curtails their regulatory and anti-inflammatory potential. Overall, these findings demonstrate that local Na^+^ availability is a promising novel regulator of immunity. Hence, the modulation of tissue Na^+^ levels bears broad therapeutic potential: increasing local Na^+^ availability may help in treating infections, while lowering tissue Na^+^ levels may be used to treat, for example, autoimmune and cardiovascular diseases.

## Introduction

In general, all extracellular body fluids are thought to readily equilibrate and display osmolalities similar to those of plasma. Traditionally, local salt storage in tissues was not considered to occur; consequently, studying local salt composition and its effect on tissues other than the kidney has been largely disregarded, although evidence to that effect already existed in 1909 [1, 2]. The generally accepted exception to this rule is of course the kidney, where large differences in electrolyte concentration are present and also required for the production of concentrated urine. For example, in the inner medulla of kidney osmolalities up to 1200 mOsm/kg can be achieved (reviewed in [3]). These increased electrolyte levels and osmolalities in this part of the kidney might especially have an impact on the biology of mononuclear phagocytes because these immune cells are known to be present in high numbers in the interstitium of the renal medulla [4, 5].

Mononuclear phagocytes consist of macrophages, monocytes and dendritic cells (reviewed in [6–8]) which play key roles in host defense due to their innate antimicrobial activity and their ability to mount and regulate adaptive immune responses (reviewed in [9–11]). However, in addition to these classical functions mononuclear phagocytes are able to adopt a regulatory phenotype that eventually leads to the resolution of inflammation and tissue repair (reviewed in [10, 12, 13]). Moreover, these versatile cells can adapt to various local tissue microenvironments and change their function according to the required needs (reviewed in [10, 12, 14, 15]). For example, tissue-derived retinoic acid polarizes peritoneal macrophages through the induction of GATA-binding factor 6 (GATA6) to support the immunoglobulin A (IgA) production of peritoneal B-1 cells which in turn play a pivotal role in early defense against pathogens [16]. Macrophages do not only protect against invading intruders, but they are also key players in orchestrating the resolution of the infection, and they have a critical function in the response to sterile ischemic insults. More specifically, in myocardial infarction, macrophages play a key role in tissue repair as well as in the activation and mobilization of stem cells (reviewed in [17]). In addition, macrophages serve as angiogenic and arteriogenic accessory cells [18–20] and thereby assist in ensuring sufficient tissue oxygenation. They are also able to support lipid handling [21, 22] and to mitigate obesity-associated inflammation [23]. These cells even orchestrate the development of brown fat and promote thermogenesis [24–26]. Therefore, it is not surprising that mononuclear phagocytes are also perfectly suited to survive and to fulfill important tasks within the very hypertonic microenvironments of the kidney.

## Salt gradients in the kidney and their impact on mononuclear phagocytes

Electrolyte concentrations found in the medullary environment of the kidney impact on the immunobiology of the renal mononuclear cell network and augment their arginase-1 (Arg1) expression, which produces urea from L-arginine [27]. Enhanced Arg1 expression of these medullary mononuclear phagocytes is linked with an anti-inflammatory phenotype [27]. This mirrors earlier findings that, in contrast to their cortical counterparts, medullary mononuclear phagocytes are weak stimulators of inflammatory T cell responses in a mouse model of crescentic glomerulonephritis [28]. Therefore, it is tempting to speculate that instead of supporting inflammatory reactions, the main function of interstitial medullary mononuclear myeloid cells is to help in the local buildup of urea solutes by breaking down L-arginine. Thereby, medullary mononuclear phagocytes might support the production of concentrated urine. In line with this notion there are two recent publications showing that these interstitial mononuclear cells indeed serve as accessory cells in the kidney’s task to handle electrolytes. Harris and coworkers demonstrated that high salt diets increase the expression of cyclooxygenase-2 in renal mononuclear phagocytes and that this renal mononuclear phagocyte-driven prostaglandin production decreased the phosphorylation and activity of the renal Na^+^–Cl^−^ cotransporter in the distal convoluted tubule in the renal cortex [29]. Using a mouse model of renin angiotensin system (RAS)-mediated hypertension, Crowley and coworkers discovered that interleukin (IL)-1 receptor signaling impairs the ability of intra-renal macrophages to facilitate tubular Na^+^ excretion [30]. Excess aldosterone [31] as well as the treatment of uninephrectomized mice with deoxycorticosterone acetate and saline to drink [32] result in overproduction of IL-1β and thereby might impact on renal Na^+^ handling. Together, these data suggest that RAS blockade might promote the macrophage’s accessory function of assisting tubular Na^+^ excretion in addition to its ability to block inflammation and fibrosis (reviewed in [33]).

Increased NaCl concentration not only modulates this homoeostatic accessory function of renal mononuclear phagocytes, it also promotes the release of inflammatory cytokines and chemokines. Studies have shown that increases in the NaCl concentration to osmolalities of approximately 500–630 mOsm/kg were sufficient to promote the release of tumor necrosis factor (TNF) [34, 35] and chemokine (C-X-C motif) ligand 2 (CXCL2; macrophage inflammatory protein-2) [34] from macrophages in the absence of any additional inflammatory priming. Ip and Medzhitov reported that an increase in the NaCl concentration by 100 mM to a total osmolality of approximately 500 mOsm/kg in lipopolysaccharide (LPS)-treated cells promoted caspase-1-dependent IL-1β- and IL-1α-release from macrophages [36], while an increase of 40 mM NaCl compared to standard cell culture conditions alone were not sufficient to promote the release of IL-1 in LPS-treated cells [37]. Mechanistically, very high salt concentrations promote the production of mitochondrial reactive oxygen species (ROS) and thereby trigger subsequent inflammasome activation [36]. Given that monosodium urate-mediated Na^+^ overload is known to trigger inflammasome activation and concomitant Na^+^ loading of macrophages [38, 39], high salt conditions might further boost the induction of urate-mediated inflammasome activation and, in general, impact on the clinical course of crystal-associated kidney diseases (reviewed in [40, 41]).

## Local salt storage in the skin of mice and men

In contrast to the prevailing notion that the kidney is the sole organ wherein electrolyte gradients can occur, there are several lines of evidence indicating that electrolyte handling in the body is much more complex than previously believed and that electrolyte gradients can occur in organs other than the kidney (reviewed in [42, 43]). Over 100 years ago, Wahlgren and Padtberg noted that following increased dietary salt intervention the skin can serve as a salt depository [1, 2]. Approximately 40 years ago, Ivanova et al. reported that huge amounts of Na^+^ can be stored in the skin and linked to an enhanced sulfation of glycosaminoglycans in rats, which might serve as a negative charge capacitor for Na^+^ [44]. A few years later, Szabo and Magyar reported that in rabbits, Na^+^ and K^+^ concentrations were higher in tissue fluids than in the serum [45]. This phenomenon has more recently also been shown in mice, where salt was found to accumulate in the skin upon a high salt diet while plasma electrolyte levels remained unchanged [46, 47].

These findings suggest that in addition to the kidney, other regulatory circuits must exist that govern electrolyte balances within tissues. We postulate that the glycosaminoglycan storage depots lead to microenvironmental domains, such that the interstitial Na^+^ concentration is substantially higher than that recorded in the plasma. While the mechanisms that drive dietary Na^+^ deposition in the skin remain elusive, it is known that the clearance of electrolytes from the skin is regulated by macrophages. A high NaCl concentration serves as a chemotactic stimulus for macrophages [48], and upon high dietary salt intake, macrophages infiltrate the skin and induce on-site vascular endothelial growth factor (VEGF)-C production [46, 49]. VEGF-C production in macrophages is directly governed by the osmoprotective transcription factor tonicity-dependent enhancer binding protein/nuclear factor of activated T cells 5 (TonEBP/NFAT5). Enhanced VEGF-C tissue levels are required to induce lymphatic hyperplasia by stimulating VEGF receptor 3 (VEGFR3) signaling [47]. This lymphatic hyperplasia is necessary for electrolyte drainage. By disturbing macrophage infiltration in the skin, VEGF-C signaling or NFAT5 signaling in macrophages interferes with the clearance of electrolytes in tissues and results in increased blood pressure [46, 49]. In line with this notion, mice displaying enhanced lymphatic vessel density display enhanced fluid drainage from peripheral tissues and are hypotensive [50]. Moreover, cyclooxygenase-2 in macrophages plays an important role in supporting macrophage-driven VEGF-C secretion and lymphangiogenesis and thereby contributes to abating salt-sensitive arterial hypertension [29].

^23^Na-Magnetic resonance imaging (MRI) technology allows for non-invasive visualization and the quantification of tissue Na^+^ stores in humans [51]. This novel technology was used to assess Na^+^ stores in various cardiovascular and kidney diseases. Primary and secondary hypertension are both linked with increases in skin Na^+^ levels [51, 52]. Hammon et al. reported that patients suffering from acute heart failure also displayed enhanced skin and skeletal muscle Na^+^ storage [53]. This was not followed by concomitant swelling of the muscle tissue, indicating osmotically inactive cellular Na^+^ storage. Upon diuretic treatment, this tissue Na^+^ accumulation was at least partially reversible [53]. This observation documents that Na^+^ storage is reversible and exchangeable.

Skin and muscle Na^+^ overload was also evident in hemodialysis patients and can be mobilized by dialysis treatment. However, the mobilization of tissue Na^+^ is impaired in older patients and patients with low VEGF-C levels [54]. Once more, this observation further underpins the concept that local regulatory circuits play an important role in tissue electrolyte balance. In this context it is tempting to speculate that increased skin Na^+^ storage in the elderly [55] might be related to a decrease in lymphatic vessel density and function during the ageing process [56]. Whether or not this age-dependent decline of lymphatic function is linked to decreased VEGF-C-levels or inhibitors of VEGF-C signaling, such as soluble VEGF-C receptors, is unknown. We further speculate that the reduced density and function of lymphatics in the elderly might impair local clearance of electrolytes from tissues and contribute to arterial hypertension.

## Skin salt storage upon infection

Local salt storage is not solely a feature of cardiovascular and kidney diseases. We recently observed that there is an unusually high amount of Na^+^ accumulation, without commensurate water retention, in the infected skin of mice bitten by cage mates and kept on a constant low salt diet [57]. Chemical analyses of the areas of infected skin demonstrated an effective total osmolyte-to-skin water ratio that was approximately 40 mM higher than concentrations in the plasma. This level resembled approximately the increase of effective osmolytes observed in mice kept on an experimental high salt diet (4 % NaCl in chow, 0.9 % NaCl in water) and suggested that the interstitial microenvironment of the infected skin is hypertonic [57]. However, the amount of effective osmolytes found in inflamed skin was considerably lower than that of the osmolytes found in the kidney medulla. In line with our findings, Schwartz et al. demonstrated that subcutaneous injection of Bacille Calmette–Guérin or Freund’s adjuvant resulted in enhanced tissue osmolalities [58]. Na^+^ accumulation in infected/inflamed tissue is not only evident in rodents, but also occurs in humans. In three studies, ^23^Na-MRI technology was used to quantify Na^+^ levels in inflamed and infected tissues in patients with multiple sclerosis (MS) and superficial streptococcal skin infections, respectively. These analyses revealed that Na^+^ is stored in inflammatory MS lesions [59, 60] and infected skin tissue [57]. Antibiotic treatment of the superficial skin infection resulted in a reduction of skin tissue Na^+^ levels [57]. These data indicate that infection/inflammation drives local salt accumulation. The regulatory circuits that drive salt accumulation upon the site of infection and/or inflammation are, however, unknown.

## A high salt level augments pro-inflammatory and antimicrobial macrophage function

We were excited by the notion that enhanced salt concentrations found at the site of infection (approx. 40 mM increase in effective osmolytes compared to plasma) might be an unappreciated beneficial strategy to ward off infections by boosting the immune system and its antimicrobial activity. Several lines of evidence support this possibility. First, changes in tissue osmolalities in zebrafish activate innate immune responses and mediate rapid wound closure and tissue repair [61–63]. Therefore, changes in tissue osmolality might represent an ancient danger signal which is advantageous because it does not require the de novo production of mediators and thereby ensures immediate delivery of the signal. Second, increases of 40 mM in effective osmolytes to a final osmolality of 380 mOsm/kg are unlikely to induce a direct effect on pathogen survival given that *Escherichia coli* can very well tolerate osmolalities of 400 mOsm/ kg [64]. Third, p38/mitogen-activated protein kinase (MAPK) and TonEBP/NFAT5 are both induced by NaCl-mediated osmotic stress and by stimulation with the pro-inflammatory bacterial cell-wall component LPS [65–67]. These observations already imply that osmoprotective and inflammatory responses might be intertwined. Also, exposure of peripheral blood mononuclear cells to increased levels of NaCl (+40 mM NaCl compared to standard cell culture conditions) enhanced the release of IL-8 in a p38/MAPK-dependent manner [68], while decreasing the osmolality below standard cell culture conditions impaired IL-8 release [69]. Similarly, increasing NaCl concentrations in cell culture media augmented inflammatory cytokine release of LPS-stimulated human peripheral blood mononuclear cells and human monocytic THP-1 cells [69–71]. Finally, the tonicity-dependent interaction between NFAT5 and nuclear factor (NF)-κB p65 subunits show a considerably enhanced nuclear factor(NF)-κB activity following the binding of NF-κB–NFAT5 complexes to κB elements of NF-κB-responsive genes [72]. After taking all these points into consideration, we hypothesized that high salt conditions do not exert a direct antimicrobial activity—rather they boost the host’s immunity and eventually help in clearing infections.

Indeed, when we performed experiments to examine this hypothesis in more detail, we observed that the inflammatory activation of macrophages stimulated with LPS in the presence of high NaCl concentrations equivalent to what had been seen in the infected skin of rodents (an increase of 40 mM NaCl) was augmented [57]. This high salt response included a marked increase in TNF release and type-2 nitric oxide (NO) synthase (Nos2)-dependent NO production, suggesting enhanced classical macrophage activation [57]. These findings were subsequently confirmed by independent research groups [73, 74]. Moreover, this enhanced pro-inflammatory activation is also present in retina pigment epithelium cells [75]. Mechanistically, high salt-boosted macrophage activation required p38/MAPK and downstream NFAT5-signaling, but it was independent of signal transducer and activator of transcription (STAT) 1-signal transduction [57]. Furthermore, this activation subsequently resulted in modified epigenetic markers. Of note, increasing the NaCl concentration by 40 mM compared to standard cell culture NaCl concentrations in the absence of LPS (i.e. NaCl alone) did not favor significant pro-inflammatory cytokine and mediator release on its own [57].

In our study [57], increasing salt availability (+40 mM NaCl compared to standard cell culture media) not only promoted macrophage activation, but it also improved antimicrobial control. High salt conditions in the absence of macrophages (+40 mM NaCl compared to standard cell culture media) did not impair growth of the pathogens, thus excluding a direct antimicrobial effect of high salt alone. Using a *Leishmania* *major* infection model, we demonstrated that boosting the anti-leishmanial activity of macrophages also required p38α/MAPK–NFAT5 signaling and subsequent *Nos2*-dependent production of leishmanicidal NO. Increasing skin Na^+^ stores by high salt diets subsequently improved cutaneous anti-leishmanial control in mice. This process required NFAT5-dependent signaling in macrophages [57]. These findings demonstrate that increases in local Na^+^ content in the skin can act in concert with tissue damage/infection as a danger signal, which in turn enhances innate immune cell activation and helps in warding off macrophage-prone skin infections.

**Fig. 1:**
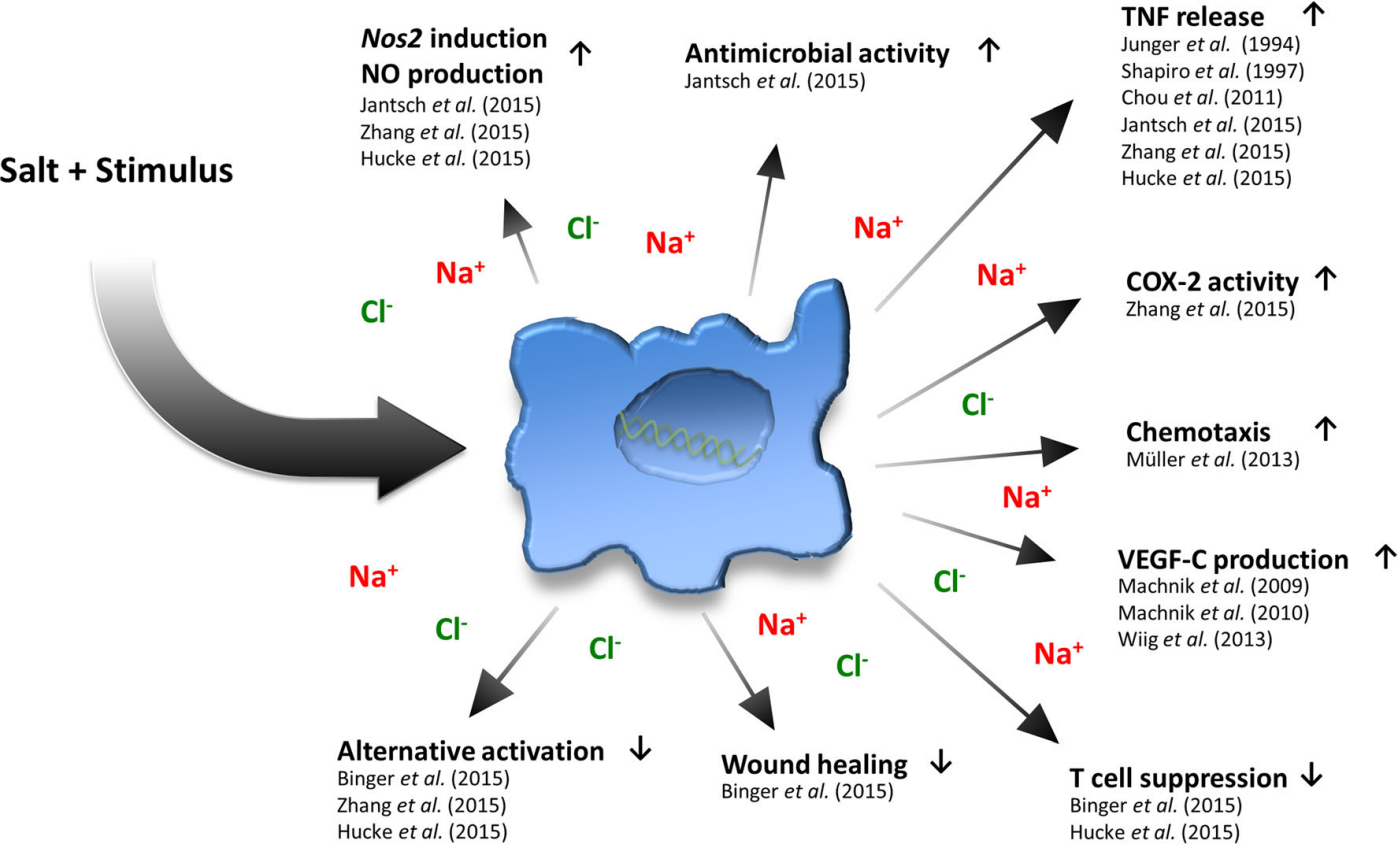
Effects of a high salt level on macrophage immunobiology.* COX-2* Cyclooxygenase-2,* NO* nitric oxide,* Nos2* type-2 NO synthase,* TNF* tumor necrosis factor,* VEGF* vascular endothelial growth factor

In contrast to favoring classical pro-inflammatory macrophage activation, high salt conditions (+40 mM NaCl compared to standard cell culture media) has been found to impair the development and functionality of IL-4- and IL-4/IL-13-driven alternative macrophage activation, which is required for tissue repair and the resolution of inflammation [73, 74, 76]. The high salt-mediated blockade of alternative macrophage activation was shown to hinge on the impairment of the ‘serine/threonine-protein kinase AKT’ and ‘mechanistic target of Rapamycin’ (mTOR) pathway, but was independent of STAT 6-signal transduction [76]. This resulted in an impaired ability of alternative activated macrophages to suppress T cell proliferation and mediate wound healing [76]. Altogether, these findings are complementary to those with pro-inflammatory classical macrophages [57], as they demonstrate that enhanced Na^+^ levels can act as a danger signal which promotes the skewing of macrophages away from an anti-inflammatory immune cell phenotype and towards a pro-inflammatory macrophage phenotype and subsequent antimicrobial control (Fig. 1).

## High salt promotes inflammatory T cell activation

Salt-induced enhancement of leukocyte function are not confined to macrophages that belong to the innate immune system, but they operate in T cells which form an essential part of the antigen-specific adaptive immune system and whose function is known to be governed by various microenvironmental cues [77]. For almost two decades it has been known that increasing NaCl conditions by approximately 40 mM boosts IL-2 expression and T cell proliferation [70, 78]. Again, this increased Na^+^ concentration mimics the levels of effective skin osmolytes observed under conditions of high salt diets and infection/inflammation, and promotes p38/MAPK signaling in T cells [79, 80]. Moreover, Loomis et al. reported that high salt conditions (+40 mM NaCl) restored IL-2 production of T cells that had been suppressed by IL-4, IL-10, transforming growth factor and prostaglandin E2 [81]. This observation suggests that increased tonicity, which is present in secondary lymphatic organs [82], favors T cell proliferation. In line with this notion, mice haplodeficient for the central osmostress transcription factor *Nfat5* displayed reduced splenocyte proliferation, impaired IgG responses after vaccination [82] and impaired cytokine production by T cells [83]. This further substantiates that high salt-induced pathways play an important role in T cell immunology.

However Na^+^ does not only favor T cell proliferation in general, but it also affects the activation/polarization of T cells. T cells differentiate into cytotoxic, helper and regulatory T cells. While the knowledge of the effect of high salt on cytotoxic T cell functions is very sparse, a substantial body of information is now available on how salt affects the helper and regulatory T cell functions. High salt conditions specifically boost the development of IL-17-producing CD4^+^ T helper cells (Th17), which are known to provide protection from infectious diseases and to worsen autoimmune diseases, such as MS [84, 85]. Mechanistically, high salt promotes the activation of Th17 cells, once again via p38/MAPK and NFAT5 [85], as well as serum/glucocorticoid-regulated kinase 1 (SGK1)-dependent signaling [84, 85]. In a mouse model of MS, high salt diets were shown to be linked to increased Th17 cell infiltration into the central nervous system and aggravated clinical outcome [84, 85]. In contrast, high salt levels have been shown to impair the functionality and development of regulatory forkhead box P3 (Foxp3)^+^ T cells (Tregs), which play a key role in self-tolerance and are dysregulated in autoimmune diseases [86, 87]. Mechanistically, this again required SGK1-signaling. High salt-induced SGK1-signal transduction has been shown to promote interferon release from Tregs, which abrogated their suppressive effects [87]. This high salt-inhibited Treg function aggravated the clinical course in a mouse model of graft versus host disease [87]. In line with the notion that high salt induces the impairment of Treg function, high salt diets accelerated allograft rejection in a mouse model, which were paralleled by a reduced frequency of Tregs [86]. Again, this was dependent on SGK1 signaling in CD4^+^ T cells [86]. Downstream of SGK1, impaired forkhead box O3 (FoxO) 1/3a signal transduction might affect the accessibility of Foxp3 to its transcriptional binding sites [87] and/or the regulation of Foxp3 expression [86]. Taken together, these studies indicate that high salt levels have differential effects on T cell activation; further studies are required to determine whether high salt conditions also impact on other T helper cell subsets.

**Fig. 2:**
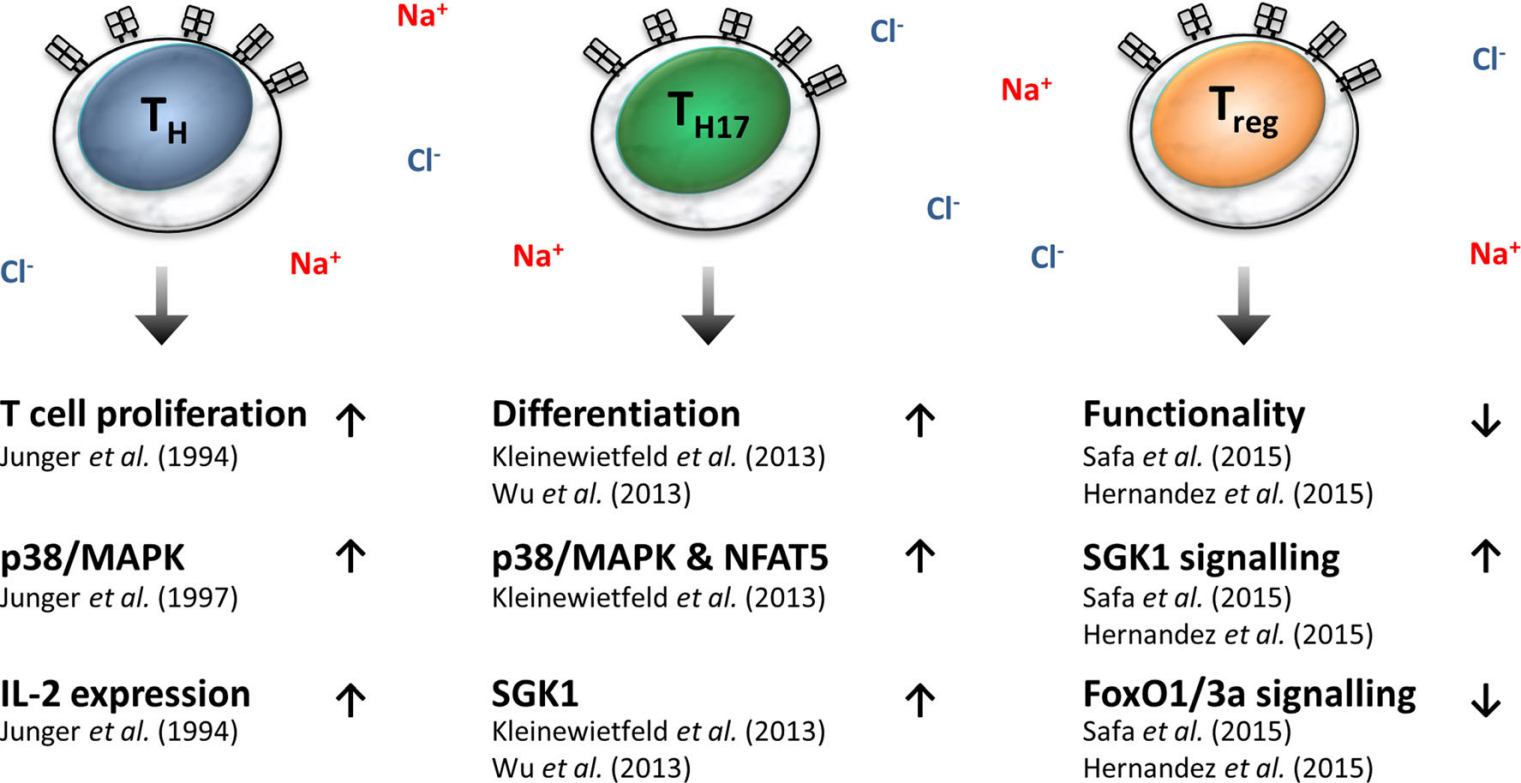
Effects of a high salt level on T cell immunobiology.* Fox01/3a* Forkhead box O3 1/3a,* IL-2* interleukin-2,* NFAT5* nuclear factor of activated T cells,* p38/MAPK* p38/mitogen-activated protein kinase,* SGK1* serum/glucocorticoid-regulated kinase 1

Importantly, while high salt diets affect Th17 and Treg activation in these preclinical models, the precise location where T cells face high salt conditions in vivo is as yet unknown. Again, T cells could encounter very salty conditions in the medulla of the kidney [88]. Other possibilities could be when T cells patrol through Na^+^-laden skin or, alternatively, high salt diets may augment Na^+^ levels in secondary lymphatic organs where T cell activation and proliferation take place. Moreover, it is possible that in vivo in addition to directly affecting T cell activation, high salt diets impact on antigen-presenting cells, such as macrophages/ dendritic cells, as well as on the ability of these cells to regulate T cell immunity, such as Th17 proliferation. In line with this notion, a recent study has demonstrated that high salt conditions favored an antigen-independent boost of T cell proliferation by enhancing pro-inflammatory macrophage activation [74]. Taken together, the available data suggest that high salt conditions favor T cell proliferation and skewing of these cells to a pro-inflammatory phenotype, while concomitantly impairing the tolerogenic functions of these cells (Fig. 2).

## Salt and other immune cells

Little information is available on other immune cells and the effect of high salt levels on their function and activation. For example, the impact of salt on the immunobiology of B cells is very limited, although it is known that in addition to producing antibodies, these cells are able to phagocytose and present antigens, as well as to fulfill important regulatory functions (reviewed in [89, 90]). To the best of our knowledge, only one report has described a role for salt-signaling molecules in B cell function: this study focuses on a guanine nucleotide exchange factor, Brx [91]. The authors demonstrate that this molecule is essential for the expression of Nfat5 in response to osmotic stress, thus transmitting the extracellular hyperosmolarity signal and enabling differentiation of splenic B cells and production of immunoglobulins [91].

In addition to some studies on B cells, there are some reports on the effect of high salt levels on granulocytes. These innate immune cells are a key component of the immediate antimicrobial innate host response. The activity of NADPH oxidase and of the myeloperoxidase-mediated ROS production are key components of their antimicrobial arsenal (reviewed in [92]). Very high concentrations of 270 mM NaCl, which might occur in the medulla of the kidney, reportedly block ROS accumulation, chemotaxis, phagocytosis and the killing capacity of neutrophils [93–96]. In line with this, it is known that very high salt conditions (as might occur in the medulla of the kidney) impair proteolytic enzymes, such as Cathepsin G [97] and elastases [98]. Increases in Na^+^ concentration by 40 mM, comparable to levels found in infected or dietary Na^+^-laden skin, did not interfere with ROS production of* N*-formyl-methionyl-leucyl-phenylalanine (fMLP)-activated granulocytes and even enhanced the elastase release of fMLP-activated granulocytes [99, 100]. Similarly, enhancing osmolarity with Na^+^/K^+^ acetate augmented the release of histamine from basophil granulocytes [101]. Whether increased salt concentrations, as found in infected and inflamed skin tissue, affect the subcellular charge balance of phagosomes is as yet unknown (reviewed in [102]). In addition, it is unclear whether enhancing salt concentrations by 40 mM affects the ROS and subsequent bleach production of infected granulocytes (reviewed in [103]) and ultimately results in enhanced antimicrobial function. NaCl injection was found to promote the infiltration of neutrophils into the peritoneal cavity, which might promote local antimicrobial defense [36]. This process might involve high salt-boosted release of the neutrophil-attracting chemokines CXCL1 and CXCL2 [73]. However, further studies are needed to understand the effect of high salt on antimicrobial granulocyte function.

## Local Na^+^ metabolism as a new regulator of immunity

Altogether, there is now substantial evidence that local Na^+^ content can act in concert with tissue damage/infection as a danger signal, enhancing proinflammatory macrophage and T cell function while dampening anti-inflammatory immune responses. Thereby, hypertonic salty microenvironments serve as a protective element against microbial invaders. Hence, an underlying principle of Na^+^ metabolism might be to strengthen the barrier function of the skin [57].

However, the mechanisms that drive local salt storage under infectious/inflammatory conditions are unknown and warrant further investigation. A detailed understanding of how tissue Na^+^ levels are regulated might open new avenues to regulate immunity and bears broad therapeutic potential. Increasing local tissue Na^+^ levels might help the host to fight intruders by enhancing the antimicrobial armory of immune cells. Interestingly, while promoting antimicrobial defense, increasing skin Na^+^ stores in *L. major*-infected mice did not promote tissue immunopathology [57] and therefore lacks an unwanted adverse side effect that usually comes with enhancements of immune-driven antimicrobial defense mechanisms [104]. Therefore, increasing tissue Na^+^ levels in the clinical context of infectious diseases might be an appropriate adjunctive strategy to fight against such infections by specifically enhancing immune-driven antimicrobial defense mechanisms without inducing an excessive inflammatory reaction that is inappropriate to ward off infection [104].

However, in the absence of microbiological invaders, Na^+^ storage occurs with dietary salt excess in animals and age in humans [52, 55]. In this context, Na^+^ storage could lead to the unintended consequence of inappropriate pro-inflammatory immune cell activation, which is supported by the findings of salt-exacerbated autoimmune encephalitis, tumorgenesis and hypertension [52, 55, 74, 84, 85, 105]. Hence, in these cases, a detailed knowledge of the regulatory circuits driving local salt accumulation and salt-dependent immune cell activation might be useful to dampen immune responses. For example, blockade of inflammation-driven salt accumulation might be used to possibly diminish inflammatory responses and thus might be used to treat hypertension, autoimmune diseases and even cancer.
